# The impact of mindfulness on suicidal behavior: a systematic review

**DOI:** 10.47626/2237-6089-2021-0316

**Published:** 2021-03-31

**Authors:** Kyara Rodrigues de Aguiar, Júlia Bierhals Bilhalva, Mariana Dias Cabelleira, Giovanna Oliveira Guimarães, Thiago Madureira, Arela Agako, Marília Silva de Souza, Luciano Dias de Mattos Souza

**Affiliations:** 1 Programa de Pós-Graduação em Psiquiatria e Ciências do Comportamento UFRGS Porto Alegre RS Brazil Programa de Pós-Graduação em Psiquiatria e Ciências do Comportamento, Universidade Federal do Rio Grande do Sul (UFRGS), Porto Alegre, RS, Brazil.; 2 Programa de Pós-Graduação em Saúde e Comportamento Universidade Católica de Pelotas Pelotas RS Brazil Programa de Pós-Graduação em Saúde e Comportamento, Universidade Católica de Pelotas (UCPel), Pelotas, RS, Brazil.; 3 Departamento de Psicologia Universidade Federal do Rio Grande Rio Grande RS Brazil Departamento de Psicologia, Universidade Federal do Rio Grande (FURG), Rio Grande, RS, Brazil.; 4 Department of Psychology, Neuroscience & Behaviour McMaster University Hamilton ON Canada Department of Psychology, Neuroscience & Behaviour, McMaster University, Hamilton, ON, Canada.

**Keywords:** Suicide, suicide ideation, suicide behavior, mindfulness, systematic review

## Abstract

**Introduction:**

Mindfulness-based interventions (MBI) have been growing progressively as treatment options in the field of mental health. Aim: To assess the impact of mindfulness-based interventions for reducing suicidal thoughts and behaviors.

**Methods:**

A systematic review was performed in December 2020 using PubMed, PsycINFO, EMBASE, SciELO, Pepsic, and LILACS databases with no year restrictions. The search strategy included the terms (‘mindfulness’ OR ‘mindfulness-based’) AND (‘suicide’ OR ‘suicidal’ OR ‘suicide risk’ OR ‘suicide attempt’ OR ‘suicide ideation’ OR ‘suicide behavior’). The protocol was registered in the International Prospective Register of Systematic Reviews (PROSPERO), CRD42020219514.

**Results:**

A total of 14 studies met all inclusion criteria and were included in this review. Most of the studies presented Mindfulness-Based Cognitive Therapy as the MBI assessed (n=10). An emerging and rapidly growing literature on MBI presents promising results in reduction of suicide risk, particularly in patients with MDD. Four studies assessing other MBI treatment protocols (Mindfulness-Based Stress Reduction; Daily Mindfulness Meditation Practice; Mind Body Awareness and Mindfulness-Based Cognitive Behavior Therapy) all demonstrated that MBI reduces factors associated with suicide risk.

**Conclusion:**

MBI might target specific processes and contribute to suicide risk reduction.

## Introduction

Mindfulness is conceptualized as a particular way of paying attention to the present moment in an intentional, non-judgmental, and non-reactive manner.^1^

Mindfulness-based interventions (MBI) have been growing progressively as treatment options in the field of mental health.^2^ Mindfulness-based Cognitive Therapy (MBCT),^3^ Mindfulness-Based Stress Reduction (MBSR),^4^ and Mindfulness-Based Relapse Prevention (MBPR) are of particular note among the most well-known MBI.^5^ Different MBI approaches have proven effective in the treatment of psychiatric disorders^6^ (e.g., anxiety, depressive, personality disorders)^7,8^ and are associated with improved outcomes of other clinical conditions (e.g., cancer, rheumatoid arthritis).^9,10^

MBSR was introduced to the field of medicine by John Kabat Zinn and was initially developed to help people manage pain and chronic conditions for which clinicians could no longer offer help.^4^ The 8-week protocol guides participants through specific meditation and movement practices to cultivate mindfulness.^4^ Since its inception, in addition to chronic pain, it has been shown to be effective in a wide variety of medical and psychological conditions and, as a result, multiple adaptations to the protocol have been developed. Teasdale et al. adapted MBSR specifically for clinical depression by incorporating components of cognitive therapy and depression psychoeducation into the curriculum (MBCT).^3^ Both protocols train participants in secular mindfulness meditation practices and metacognitive coping skills that help them manage physical feelings of anxiety (MBSR) or triggers that can precipitate a relapse into depression (MBCT). Based on the MBSR and MBCT framework, Marllat et al. developed a program for addictive behavior (MBRP).^5^ Their protocol also consists of 8 weekly sessions that integrate cognitive-behavioral relapse prevention skills with mindfulness practices.

Suicidal thoughts and behaviors are important public health concerns around the world,^11^ although the prevalence appears to be higher in lower-middle-income countries such as Brazil.^12^ In this context, it is imperative to identify feasible preventive treatments. MBI can promote changes in neuroplasticity^13^ and can facilitate the process of emotional regulation^14^ which is a key component of suicidal risk in some disorders.^15^ There is evidence that MBI have a positive effect on mediating and precipitating factors of suicidal behavior^16,17^ which could potentially make them a preventive intervention for suicide risk.

However, the literature on MBI and suicide risk is mixed. Although there is possibly a relationship between MBI and a lower incidence of suicidal thoughts and behavior, a recent review has identified suicidal behavior as a possible adverse effect of mindfulness.^18^ In contrast, three other reviews suggest MBI is effective in reducing suicide risk.^19-21^ However, one of these reviews did not follow a systematic approach for the literature review process^20^ and only half of the articles included in each of the two most recent systematic reviews were in common between them because of use of different selection criteria,^19,21^ making it difficult to draw conclusions. Thus, the aim of the current systematic review is to assess the impact of MBI for reducing suicidal thoughts and behaviors using a more comprehensive search strategy.

## Methods

The Preferred Reporting Items for Systematic Reviews and Meta-analysis (PRISMA) guidelines were followed for the present review.^22^ The protocol was registered on the International Prospective Register of Systematic Reviews (PROSPERO) under ID CRD42020219514, on December 14, 2020.

### Eligibility criteria

- Population: Any study that reported measures of suicidal ideation or suicide attempts in participants from clinical or non-clinical samples at any stage of development, without excluding any physical or psychiatric conditions.
- Intervention: Studies using psychosocial or psychotherapeutic interventions based on mindfulness were included.
- Comparators: Any study that reported other forms of psychotherapy, psychopharmacological, or psychosocial interventions, treatment as usual (TAU), or without treatment (waiting list) as control was included. Pre-post intervention studies without control groups were also included.
- Outcome: Studies that assessed suicide risk (suicidal thoughts or behavior) as an independent variable through either a question/item or scale for interview both before and after the proposed MBI were included.
- Design of the study: Studies characterized as randomized controlled trials (RCT), including cluster RCTs, non-randomized controlled trials (non-RCT), studies using a historically controlled design or uncontrolled longitudinal design, and pre-post intervention (quasi-experimental) studies were included.
- Language: Studies published in English, Spanish or Portuguese were included.

### Information sources

We searched PubMed, PsycINFO, EMBASE, SciELO, Pepsic, and LILACS with no year restrictions for articles up to December 2020.

### Search strategy

Our search strategy was designed to be broadly sensitive to ensure a comprehensive search. The following search terms were used: ‘suicide’ OR ‘suicidal’ OR ‘suicide risk’ OR ‘suicide attempt’ OR ‘suicide ideation’ OR ‘suicide behavior’ to capture outcomes. For the intervention, we used the terms ‘mindfulness’ OR ‘mindfulness-based’. The search: (‘mindfulness’ OR ‘mindfulness-based’) AND (‘suicide’ OR ‘suicidal’ OR ‘suicide risk’ OR ‘suicide attempt’ OR ‘suicide ideation’ OR ‘suicide behavior’) was conducted on all of the electronic research databases mentioned above.

### Selection process

**Figure 1:**
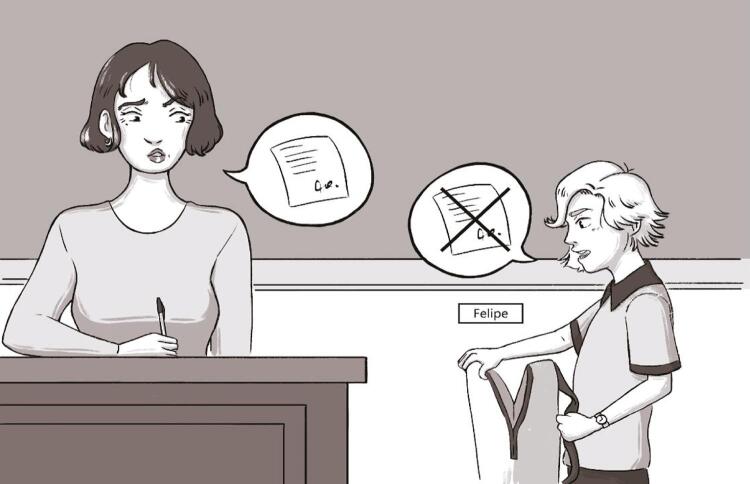
PRISMA flowchart illustrating the systematic review process.

All abstracts of articles selected in the searches of each electronic database mentioned above were double screened by two reviewers who evaluated each of the studies against specific criteria, depending on study design. In cases of disagreement, a third author read the full text and discussed each article with the reviewers until a consensus was reached. Additionally, the references from other reviews on the subject^19-21^ were scanned in order to identify potential additional articles (Figure 1).

### Data collection process

The Rayyan QCRI platform was used to remove all duplicated documents and run the first screening based on title and abstracts, done by two independent reviewers.^23^

The platform automatically identifies duplicates and following this process, one of the researchers (KRA) reviewed the articles that were flagged and manually deleted duplicates. Subsequently, using the “blinding on” option, two independent researchers (KRA, LDMS) accessed the platform and carried out screening of articles by reading the title and abstract. Lastly, the full texts of articles were reviewed (KRA, MS, JBB, GOG, MDC).

### Data extraction

The primary outcome assessed was suicidal ideation. We also included secondary outcome measures of suicidal behavior, defined as suicide attempts and suicide deaths.

As we were interested in the effects of the interventions, we reported effects at post intervention as well as all possible follow-up periods (when available) for each outcome. For each article we collected information about the first author, year of publication, study aim, design, number of participants, sample selection process, specific sample characteristics, description of mindfulness intervention, comparison group, instruments used to measure suicide risk, main results, and results regarding suicide ideation or behavior.

### Study risk of bias and quality assessment

For each paper included in this study, two authors (LDMS, AA) used the Cochrane risk of bias tool to assess the quality and risk of bias for the clinical trials initially selected^24^ or used the NHLBI tool to assess the quality of pre-post intervention studies without control groups.^25^ In cases of disagreement between evaluators, a third author was called in to break the impasse.

## Results

The literature search yielded 630 studies. Of these, 213 were duplicates and 396 studies were excluded because the titles and abstracts were not relevant to the research topic, leaving 21 potentially eligible studies for which the full text was reviewed. In addition, we hand-searched the references of the studies included and found 2 additional studies to include. At this stage, 9 studies did not meet the inclusion criteria. A total of 14 studies met all inclusion criteria and were included in the systematic review (Figure 1). Of these, 7 were randomized clinical trials (2 of which were pilots) and 7 were pre and post-intervention studies with no control group (quasi-experimental studies). All studies employed a convenience sampling strategy.

Most of the studies presented MBCT as the MBI (n = 10). For this reason, we split the results description into two sections according to the MBI used to better interpret its impact on suicide risk. Table 1 shows an overview of the studies included. Publication dates ranged from 2007 to 2019, and 6 studies were conducted in the United States of America. Total sample size ranged from 10 to 194. Regarding the assessment of suicide, four of the studies used the Beck Scale for Suicide Ideation (BSSI) and another one used a modified version of this instrument. Suicidal thoughts, ideation, or cognitions were part of the outcome of all papers included. None of the studies presented suicide death as outcome. Suicide attempts or suicidal behaviors were part of the outcome of at least 6 studies (Table 1).

**Table 1:** Main features of studies included in the systematic review

Study	Country	Design	Sample	Gender	Age	MBI	Control group	Suicide outcome measure	Main outcome related to suicide
Nabipour et al.,^26^ 2018	Iran	Randomized clinical trial	30 cancer patients	There is no information about gender of the sample	There is no information about age	MBCT	Unclear	BSSI	Reduction in suicidal thoughts in MBCT group.
Britton et al.,^27^ 2014	USA	Randomized clinical trial	100 children	45.5% were female	Mean age of 11.8 years	Daily mindfulness meditation practice lasting 6 weeks.	African history course with a matched experiential activity	Item-18 and 91 of Youth Self Report were combined to make a suicidal ideation/behavior critical item subscale	Greater reductions in development of suicidality in the MBI group.
Serpa et al.,^28^ 2014	USA	Quasi-experimental	78 veterans	89% were male	Mean age was 60	MBSR 9 weekly sessions including seated and walking meditations, gentle yoga, body scans, and discussions of pain, stress, and mindfulness.	There was no control group	Item 9 of PHQ-9	The frequency of suicidal ideation decreased by almost half, from 24% to 12%.
Forkmann et al.,^29^ 2014	The Netherlands	Randomized clinical trial	130 veterans	79% were female in intervention group and 73% were female in control group	Intervention group mean age was 44.6 and control group mean age was 43.2	MBCT Meditation exercises combined with cognitive behavioral techniques. Total of 20 hours.	Waiting list	Respective item from the self-rating form of the Dutch version of the Inventory of Depressive Symptoms	Post-hoc tests showed a significant reduction of suicidal ideation in the MBCT-group, but not in the waiting list control-group.
Forkmann et al.,^30^ 2016	Germany	Randomized clinical trial	106 outpatients	65.7% were female in the TAU group, 63.9% were female in the CBASP group, and 58.2% were female in the MBCT group	Mean age was 50 years in the TAU group, 50.2 years in the CBASP group, and 48.4 years in the MBCT group	MBCT Individual pre-class interview and eight weekly 2.5-hour group sessions. Total number of hours: 20.	CBASP - consisted of two individual treatment sessions and eight weekly 2.5-h group sessions. TAU	Item 3 from the HAMD and item 9 of the BDI	MBCT and CBASP reduced suicidal symptoms if measured with the HAMD suicide item. Neither MBCT nor CBASP showed an incremental effect on the reduction of SI as assessed with the BDI suicide item compared to TAU.
Chesim et al.,^31^ 2016	USA	Quasi-experimental	10 outpatients	They were mostly female (80%)	They ranged in age from 18 to 64 years (mean 41.7)	MBCT developed to prevent suicidal behavior. The intervention lasted 9 weeks (SPI individually + 8 sessions of weekly group MBCT-based treatment). Total of 16 hours.	No control group.	Leiden Depression Sensitivity Index - Revised	Participants evidenced significant reductions in hopelessness/suicidality from pre to post-MBCT-S treatment.
Lu et al.,^32^ 2019	China	Randomized controlled trial	49 children	25.4% of participants were female	The average age of participants was 11.7	Based on MBCT with practices from MBSR. 8 weeks with 1 hour group sessions once a week and homework exercises. Total of 8 hours.	Waiting list	PANSI	The mindfulness-training group showed a significant decrease in suicide ideation.
Barnhofer et al.,^33^ 2009	United Kingdom	Randomized controlled trial	28 patients	About two thirds were women	Most participants were middle-aged	MBCT with some minor alterations to address suicidality plus TAU. Eight weekly classes of 2h duration plus homework for about an hour per day for six days a week. Total of 16 hours.	TAU	BSSI	Neither analyses by intent-to-treat nor of the per-protocol sample yielded significant effects.
Barnhofer et al.,^34^ 2015	United Kingdom	Randomized controlled trial	194 patients	74% were women	Mean age was 43.7 years	MBCT, except that they placed greater emphasis on patterns and thoughts associated with suicidality. Eight weekly classes of 2-hour sessions Total of 16 hours.	TAU CPE that comprised all elements of the MBCT program except for the experiential cultivation of mindfulness through meditation practice	BDI-II Suicidal Cognitions Scale	Participants in the MBCT group showed a general reduction in suicidal cognitions.
Raj et al.,^35 ^2019	India	Quasi-experimental	30	50% respondents were female	Mean age was 14 years	MBCT. 12 sessions including assessments in the first and the last session. Sessions once a week, 45 minutes to an hour duration. Total amount of hours between 7h30 to 10 hours.	No control group	Modified version of the BSSI	Scores on the Modified Scale for Suicidal Ideation decreased in the post-test.
Kenny et al.,^36^ 2007	Australia	Quasi-experimental	76 depressed patients and outpatients	20 were male and 59 were female.	They were aged between 17 and 61 years	MBCT 8 weekly classes of 2 hour sessions, up to an hour of which was spent in meditation practices. Homework involved around 1 h per day of meditation or yoga, and other related formal and informal practices for the 8 weeks. Total number of hours: 16.	No control group	BDI-II	They examined which BDI items significantly differed at pre-treatment for those who did and those who did not report suicidality.
Chesin et al.,^37^ 2015	USA	Quasi-experimental	16	83% were female	They were aged between 18 and 64 years (mean=41.7)	MBCT to prevent suicidal behavior. Nine weekly classes of approximately 2-hour sessions. Session one was provided individually. Sessions two through nine were delivered in a group format. Beginning in session two, participants were asked to complete 20 min of formal mindfulness meditation practice three times per week between sessions. Total of 24 hours.	No control group	CSHF BSSI	Completers evidenced significant reductions in suicidal ideation, as measured using the BSSI, and in depression, as measured using the BDI-II, pre to post-MBCT-S treatment. In the intent-to-treat analyses, reductions in suicidal thoughts trended toward significance.
Le & Gobert,^38^ 2015	USA	Quasi-experimental	8 youths	5 were male	Mean age 17 years	Adaptation/translation of a mindfulness curriculum for cultural relevancy with Native American traditions and spiritual practices. The mindfulness classes comprised four sessions per week, 55 min per session, over 10 weeks with weekly homework assignments. Total of 36 hours.	No control group	Item 9 of PHQ-9	At pre-test, 44 % reported several days to more than half the days of thinking that they thought that they were better off dead or had thoughts of hurting themselves in some way, while at post-test, 100% responded not at all to the item.
Miklovitz et al.,^39^ 2009	USA	Quasi-experimental	16 outpatients	16 were female and 6 were male	Mean age 40.6 years	MBCT Mindfulness meditation strategies and traditional cognitive-behavioral techniques to address the mode in which negative thoughts and feelings and emerging manic symptoms are processed. Total of 16 hours.	No control group	BSSI	Reductions were observed in suicidal ideation.

BDI = Beck Depression Inventory; BSSI = Beck Scale for Suicidal Ideation; CBASP = Cognitive Behavioral Analysis System for Psychotherapy; CPE = Cognitive Psychoeducation; CSHF = Columbia Suicide History Form; HAMD = Hamilton Depression Rating Scale; MBCT = Mindfulness-Based Cognitive Therapy; MBI = mindfulness-based interventions; MBSR = Mindfulness-Based Stress Reduction; PANSI = Positive and Negative Suicide Ideation; PHQ-9 = 9-Item Patient Health Questionnaire; TAU = treatment as usual.

### The impact of mindfulness on suicide risk: evidence from MBCT

The systematic review included 10 studies assessing whether MBCT reduces suicide-related outcomes. Nine of them showed that MBCT reduces factors associated with suicide risk.^20,26,29,30,32-34,36,37,39^ Two studies employed Mindfulness-Based Cognitive Therapy for Suicide (MBCT-S) including specific contents about suicide ideation and behaviour.^20,37^

In a study by Barnhofer et al. investigating the effects of MBCT in patients currently symptomatic with chronic Major Depressive Disorder (MDD) and with history of suicide risk, participants were randomly allocated to receive MBCT plus treatment-as-usual (TAU) or TAU alone.^26^ Suicidal ideation was measured using the BSSI. Neither analyses by intention to treat (p = 0.50) or of the completers subset (p = 0.47) showed significant effects on BSSI scores. In this pilot study, the MBCT was not able to reduce suicidal ideation.

Another study included veterans recruited from outpatient mental health care with MDD in partial remission after at least one episode of MDD.^29^ Suicidal ideation was measured using the respective item from the self-rating form of the Inventory of Depressive Symptoms. The focus was on stable residual symptoms in the context of a previous depressive episode. In this study, participants were also randomized to a treatment arm TAU plus MBCT or a TAU plus waiting list arm. Post-hoc tests showed a significant reduction in suicidal ideation in the MBCT-group (p = 0.008), but not in the waiting list control-group (p = 0.41). These results suggest that MBCT may reduce suicidal ideation in patients with residual depressive symptoms. However, the impact of MBCT on suicidal ideation may be partly mediated by reduction in worry and not by change in depression, rumination, or mindfulness skills. Further, another study included an outpatient sample, although participants in the sample had Persistent Depressive Disorder.^34^ In this study, patients were randomly assigned to either TAU or to receive – in addition to TAU – either MBCT or Cognitive Behavioral Analysis System of Psychotherapy (CBASP). Suicide risk was measured with item 3 of the Hamilton Depression Rating Scale (HAMD) and item 9 of the Beck Depressive Inventory (BDI). The aim of the present investigation was to examine the effects of group MBCT and CBASP, compared to a TAU condition, on suicide ideation in chronic depressed patients, while controlling for changes in other depressive symptoms. Baseline scores on the HAMD suicide item did not differ between treatment groups. However, significant differences between treatment groups at baseline were found for the BDI suicide item (p = 0.02), with significantly lower scores on the BDI suicide item in the CBASP group than in the MBCT group. Paired sample tests revealed that both MBCT (p = 0.04) and CBASP (p = 0.00) reduced suicidal symptoms if measured with HAMD. However, suicide measured with the BDI suicide item was reduced by MBCT (p = 0.03), but not by CBASP. TAU did not show any effect on suicide in the pre-post evaluation, either according to HAMD or BDI scores. On the other hand, neither the MBCT (p = 0.72) nor the CBASP (p = 0.32) showed any incremental effect in reducing suicide ideation as assessed with the BDI suicide item, when compared to TAU. According to the HAMD, treatment condition (MBCT p = 0.02; CBASP p = 0.001) had a significantly larger effect on suicide ideation than TAU. Additionally, when compared to each other with the BDI, with MBCT as a reference condition, CBASP showed a significantly smaller effect on suicide (p = 0.03) compared to MBCT. These results showed that both MBCT added to TAU and CBASP added to TAU have an additional effect on suicidality, specifically when measured with the HAMD suicide item.

Chesin et al.^20^ conducted a pre-post quasi-experimental project seeking to test changes in cognitive functioning after an MBCT-S intervention in outpatients who had a 6-month history of attempted suicide or active suicidal ideation plus suicidal ideation at the beginning of the study. Data on history of suicide attempts were collected using the Columbia University Suicide History Form and the hopelessness/suicidality subscale of the Leiden Depression Sensitivity Index - Revised (LEIDS-R) was used to measure cognitive reactivity to hopelessness/suicidality. Their findings showed significant reductions in rumination and cognitive reactivity to hopelessness/suicidality post MBCT-S treatment (p < 0.01). However, changes in cognitive functioning were not related to changes in suicidal ideation during treatment. This study suggests that the improvements observed in cognitive functioning of hopelessness/suicidality were not simply the result of improved mood with treatment, since these improvements were also not correlated with improvements in depression during MBCT-S. Another study^32^ assessed suicidal cognitions investigating the impact of MBCT, Cognitive Psychoeducation (CPE), and TAU in 194 patients with a history of suicidal depression in a randomized controlled trial. They reported a significant difference between levels of improvement of suicidal cognitions in the group who had received MBCT (p < 0.001) and groups who had received treatments that did not include training in mindfulness. Furthermore, despite the fact that levels of depressive symptoms remained relatively unchanged, participants in the MBCT group showed a general reduction in suicidal cognitions.

Another study, conducted with patients who had endorsed current suicidal ideation and had a history of serious suicidal ideation or a suicide attempt within the past 6 months, showed significant post-treatment reductions in suicidal ideation, which was measured with the BSSI before and after the MBCT-S (p = 0.02). However, considering the data from dropout patients, the intent-to-treat analysis presented a trend towards significance for suicidal thoughts (p = 0.07).^38^

A study conducted with the objective of testing the effectiveness of MBCT in depressive patients analyzed a sample of currently actively depressed participants who had not responded fully to standard treatments.^27^ This study assessed participants who met DSM IV criteria for MDD, Bipolar Disorder in the mood depressive episode (MDE) phase or Persistent Depressive Disorder with/without suicidal ideation as part of their symptoms. They compared the pre-treatment BDI scores of patients who reported thoughts of death and suicide and those who did not report such thoughts. The average BDI for suicidal patients in pre-treatment was significantly higher than the scores for patients without suicidal thoughts (p = 0.003). Both groups’ BDI scores had decreased after treatment. The results illustrate that although individuals experiencing suicidal ideation started MBCT with a greater severity of depression, there were no differences in their pattern of response to MBCT. Although the authors did not present any absolute data about suicidal ideation at the post intervention assessment, they described in the results section that there was a significant reduction in the suicide item post-treatment in addition to four other items (sadness; lack of pleasure; guilt; and loss of interest).

Another study including patients with bipolar disorder examined the feasibility of and benefits associated with an 8-week MBCT intervention and found a reduction in symptom scores from pre to post treatment in BSSI scores (Pretreatment M [SD] 4.05 [5.69] - Posttreatment M[SD] 1.87 [2.02] (d = 0.51, SE = 0.31). However, only 22 cases were involved in this study and no statistical methods were used. In this study, MBCT seemed to be a promising treatment option for bipolar disorder, particularly for managing subthreshold depressive symptoms.^40^

Two studies investigated the impact of MBCT in specific samples.^30,36^ One study selected cancer patients (women) who were randomly and equally divided into a control group and an experimental group.^36^ Suicidal ideation was measured using BSSI. The goal of this study was to evaluate the effectiveness of MBCT for reducing suicidal thoughts and death anxiety of patients with cancer. The MBCT significantly reduced suicidal thoughts and death anxiety in the experimental group (p < 0.01).

Another study examined Chinese children left-behind by one or both parents to examine the effectiveness of MBI on suicide ideation and other mental health outcomes.^30^ The Positive and Negative Suicide Ideation (PANSI) inventory was used to measure suicide ideation. In this study, participants were randomized to a MBI training group or a waiting list control group. The MBI training group was based on an MBCT protocol. As some of the protocol was designed for working with adults and depression, it was adapted for the present sample, replacing such sessions with MBSR protocol practices and creating MBCT-based game sets. When compared with the waiting list control group, the mindfulness training group showed a significant reduction in suicide ideation following the intervention (p = 0.049). These findings indicate that MBCT can reduce suicidal ideation in the aforementioned groups.

### The impact of mindfulness on suicide risk: evidence from heterogeneous MBI

The systematic review included four studies assessing different MBI treatment protocols.^27,28,35,38^ All of them showed that MBI reduces factors associated with suicide risk. The interventions ranged from MBSR^39^ through Daily mindfulness meditation practice,^33^ Mind Body Awareness,^38^ and Mindfulness Based Cognitive Behavior Therapy.^28^

One study including veterans at a large urban Veteran Health Administration (VA) with the aim of presenting results on the quality of the effects of an MBSR^41^ course on several health outcomes showed that the frequency of suicidal ideation decreased by almost half after the intervention (p = 0.049).^39^ The study used item 9 from the 9-Item Patient Health Questionnaire (PHQ-9) to measure suicidal ideation. Le et al.^32^ aimed to verify whether a mindfulness-based prevention intervention, Mind Body Awareness,^4^ could be translated and implemented in a Native American youth population.^35^ One group of eight youths participated in a 9-week pilot of the intervention. Suicidality was also assessed through the PHQ-9. At pre-treatment, 44% of participants reported they were better off dead or had thoughts of hurting themselves in some way “several days” to “more than half the days.” At post-treatment, 100% of participants responded “not at all” to the item. No statistical method was employed to verify significant differences between the assessment times. These finds suggest that MBI for indigenous communities can be culturally appropriate and sustainable in real world settings.

Concerning adolescents with Depression and Suicidal Ideation, a pre-post study with the objective of analyzing the effectiveness of the Mindfulness Based Cognitive Behavior Therapy intervention and exploring the potential usefulness of mindfulness techniques showed that suicidal ideation scores decreased post-treatment.^28^ Suicidal ideation was measured using the Modified Scale for Suicidal Ideation (MSSI). The Mindfulness Based Cognitive Behavior Therapy sessions included cognitive behavior therapy techniques (psychoeducation, emotional regulation skills) and techniques of mindfulness (exercises and application of its understanding in the patient’s life). This study was excluded from the MBCT report because it emphasizes psychoeducation as a CBT technique and did not present inquiry as a mindfulness technique.

Finally, another study examined effects of mindfulness when applied to a group of children.^33^ Sixth-grade students were divided into four classrooms that were matched in aptitude, learning style, maturity, social characteristics, and gender. Each fall, two classrooms were randomly assigned to the meditation intervention, while the remaining two classrooms were assigned to an active control condition. This study sought to examine the effects of a mindfulness meditation intervention on standard clinical measures of mental health and affect in high school children. The meditation condition integrates traditional “third person” didactic, knowledge-based learning, with critical first person experiential learning: (1) breath; (2) awareness; and (3) body sweeps. The third person (didactic) portion of the active control condition was a 6-week curriculum on the history of ancient Africa (including Egypt). To coincide with the first person component of the meditation condition, the control condition included a non-didactic, experiential and new activity that matched the didactic content of the class: building a life-size three-dimensional model of a Pharaoh’s tomb. The Youth Self Report (YSR) was used to evaluate ideation or self-harm. The study showed that in the post-intervention, 10.4% of controls reported suicidal ideation or self-mutilation, while no meditators reported such behavior or ideation (p = 0.005).

### Quality assessment

Ratings on quality of evidence of the studies included indicated a fair/good quality for reduction of suicide risk post intervention in most of the pre-post intervention studies, with the exception of items relating to blind assessment of the participants and multiple times outcome measures (Table 2). Cochrane risk of bias tool assessment of the 7 clinical trials included showed low risk of bias for most of the items with exception of blinding of participants and personnel item (Table 3).

**Table 2:** NHLBI Tool Quality Assessments of Pre and Post-Intervention Studies

Paper	1	2	3	4	5	6	7	8	9	10	11	12
Kenny et al.,^36^ 2007	Good	Good	Good	Good	Good	Good	Good	Poor	Good	Good	Poor	Good
Miklowitz et al.,^39^ 2009	Good	Good	Good	Good	Fair	Good	Good	Poor	Poor	Poor	Poor	Good
Serpa et al.,^28^ 2014	Good	Fair	Good	Fair	Good	Good	Good	Poor	Poor	Good	Poor	Good
Chesin et al.,^37^ 2015	Good	Good	Good	Good	Good	Fair	Good	Poor	Good	Good	Poor	Good
Le & Gobert.,^38^ 2015	Good	Poor	Good	Poor	Poor	Good	Good	Poor	Good	Poor	Poor	Good
Chesin et al.,^20^ 2016	Good	Good	Good	Good	Good	Good	Fair	Poor	Good	Good	Poor	Good
Raj et al.,^35^ 2019	Good	Good	Good	Good	Good	Good	Good	Poor	Fair	Good	Poor	Good

1. Was the study question or objective clearly stated?

2. Were eligibility/selection criteria for the study population prespecified and clearly described?

3. Were the participants in the study representative of those who would be eligible for the test/service/intervention in the general or clinical population of interest?

4. Were all eligible participants that met the prespecified entry criteria enrolled?

5. Was the sample size sufficiently large to provide confidence in the findings?

6. Was the test/service/intervention clearly described and delivered consistently across the study population?

7. Were the outcome measures prespecified, clearly defined, valid, reliable, and assessed consistently across all study participants?

8. Were the people assessing the outcomes blinded to the participants’ exposures/interventions?

9. Was the loss to follow-up after baseline 20% or less? Were those lost to follow-up accounted for in the analysis?

10. Did the statistical methods examine changes in outcome measures from before to after the intervention? Were statistical tests done that provided p values for the pre-to-post changes?

11. Were outcome measures of interest taken multiple times before the intervention and multiple times after the intervention (i.e., did they use an interrupted time-series design)?

12. If the intervention was conducted at a group level (e.g., a whole hospital, a community, etc.) did the statistical analysis take into account the use of individual-level data to determine effects at the group level?

**Table 3:** Cochrane risk of bias tool for assessment of study quality

Paper	Random sequence generation	Allocation concealment	Blinding of outcome assessment	Blinding of participants and personnel	Incomplete outcome data	Selective reporting	Other bias
Barnhofer et al.,^33^ 2009	Low	Unclear	Unclear	Low	Low	Low	N/A
Barnhofer et al.,^34^ 2015	Unclear	Low	High	Unclear	Unclear	Low	N/A
Britton et al.,^27^ 2014	Low	Low	Low	Low	Low	Low	Potential bias from committee of educators
Forkmann et al.,^29^ 2014	Low	Low	High	High	Low	Low	N/A
Forkmann et al.,^30^ 2016	Low	Low	Low	Unclear	Low	Low	Potential bias from exclusion of Borderline Personality Disorder patients
Lu et al.,^32^ 2019	Unclear	Unclear	High	Unclear	High	Low	Participants were students who didn’t have classes at the time of group
Nabipour et al.,^26^ 2018	High	Unclear	High	Unclear	Unclear	Unclear	No demographic information on participants

## Discussion

There is an emerging and rapidly growing body of literature on MBI that demonstrates promising results for reduction of suicide risk. For MBCT, specifically, there are multiple good quality studies supporting its impact, reducing suicide risk, especially in populations with MDD. Other studies of MBI in general also support its use for reducing suicide risk.

Only one study presented no effect of MBI on suicide risk.^33^ Another study found conflicting results depending on how suicide ideation was measured.^30^ Both studies pointed out that only a small subset of participants had presented with suicidal ideation at the baseline assessment. These results suggest that a possible floor effect could be present that may have influenced the results of the statistical analysis. Furthermore, the way that suicidal ideation was assessed in these two studies was also highlighted as a potential explanation for the results observed.^34^ The larger variance in responses and use of more sensitive terminology in item 3 of the HAMD may have facilitated observation of a statistically significant difference. This is particularly important when compared with item 9 of the BDI,^34^ which was used as an outcome measure in some of the studies above, but which doesn’t allow for as much variance in responses and uses different terminology.

MBI is associated with several positive benefits that might mediate this effect on suicide risk. MBI reduces factors such as impulsivity and depressive symptoms,^12,18^ which are associated with a higher risk of suicide. Furthermore, it also improves various cognitive processes like executive functioning^20^ and attention^20,27^ and increases meta-awareness^42^ and self-consciousness.^42^ The hypothesis that MBI training increases formal practice of better cognitive processes and a less symptomatic lifestyle and, consequently, leads to lower suicide risk seems very plausible. Learning mindfulness skills is an important mediator in reduction of depressive symptoms.^39^ However, the mechanisms of change involved in this process are as yet unknown. Changes in specific processes, like worry, appear to mediate the effect of MBI intervention on suicidal ideation and depressive symptoms.^29,43,44^ The repetitive thinking worry process is considered a proximal risk factor for suicide behavior and is frequently associated with suicide ideation.^44-46^ It was surprising that MBI did not improve rumination,^36^ as formal mindfulness meditation has been associated with improvements in this construct.^47^ Furthermore, worry and rumination are highly correlated processes which may suggests that there might be something specific to rumination in the context of suicidal ideation that may need further exploration.^48^ Moreover, MBI improves an individual’s ability to delineate the prodromal signs and symptoms related to a past suicidal crisis possibly due to memory process and meta-awareness improvements.^42^ MBI might target specific processes and collaborate with suicide risk reduction. However, more clinical studies of potential mediators are necessary.

We believe that the most important point that should be highlighted in our work is our research strategy. This is designed to be broadly sensitive in order to ensure a comprehensive search. However, there are limitations to this review that should be acknowledged. First, there might be a possible publication bias, considering that most of the studies in the literature provided positive results which demonstrate the effectiveness of MBI in reducing the risk of suicide. It is possible that studies with null results might not have been published and therefore not included in this article.

The second limitation is the variability among studies regarding the definition of suicide risk as well as the instruments used and the criteria evaluated with them. Most studies made exclusive use of self-report measures of suicide ideation, excluding variables such as suicidal behaviors, non-suicidal self-injury, and suicidal attempts. Future research might benefit from including suicide attempts as a potential outcome in larger samples as well as observed suicidal behavior over the long term.

Lastly, the heterogeneity in the format of mindfulness protocols makes it difficult to synthesize findings. Although, it is important to adapt psychosocial interventions to their audiences, the cultural aspect of patient samples might produce different results.^32^ Furthermore, the mindfulness protocols were different in terms of duration (number of sessions, time of each session), practices and exercises used, and intervention models (group x individual), and protocols had different targets. All this variability between studies makes it difficult to synthesize results and draw formal conclusions regarding efficacy and effectiveness. Further research is needed in terms of which aspects of the current MBI protocols are most effective for reducing suicidal risk.

It is thus worth highlighting that this research may be important for development of a more robust MBI protocol which could then be employed and researched as a possible preventative measure of suicide risk. To contribute to this topic, we conducted a rigorous search including recent data (December 2020) in a large number of databases, following the PRISMA guidelines, registered the protocol on PROSPERO, and assessed the quality of the studies included.

Overall, it has been demonstrated that MBI is a feasible and effective treatment for reducing suicide risk, and MBCT in particular, when used with individuals with mood related disorders. Although more research is necessary into MBI as a preventative tool for suicide risk, the current results are promising.
